# GlycA, a Biomarker of Low-Grade Inflammation, Is Increased in Male Night Shift Workers

**DOI:** 10.3390/metabo12121172

**Published:** 2022-11-24

**Authors:** Daniele Bizzarri, Martijn E. T. Dollé, Bette Loef, Erik B. van den Akker, Linda W. M. van Kerkhof

**Affiliations:** 1 Biomedical Data Sciences, Leiden University Medical Center, 2333 ZC Leiden, The Netherlands; 2 Center for Health Protection, National Institute for Public Health and the Environment, 3720 BA Bilthoven, The Netherlands; 3 Center for Nutrition, Prevention and Health Services, National Institute for Public Health and the Environment, 3720 BA Bilthoven, The Netherlands; 4 Intelligent Systems, Pattern Recognition and Bioinformatics, Delft University of Technology, 2628 XE Delft, The Netherlands

**Keywords:** night shift work, circadian rhythms, metabolomics

## Abstract

Sustained night shift work is associated with various adverse health risks, including an increased risk of cardiovascular disease, type II diabetes, and susceptibility to infectious respiratory diseases. The extent of these adverse health effects, however, seems to greatly vary between night shift workers, yet the underlying reasons and the mechanisms underlying these interindividual differences remain poorly understood. Metabolomics assays in the blood have recently gained much attention as a minimally invasive biomarker platform capturing information predictive of metabolic and cardiovascular diseases. In this cross-sectional study, we explored and compared the metabolic profiles of 1010 night shift workers and 1010 age- and sex-matched day workers (non-shift workers) from the Lifelines Cohort Study. The metabolic profiles were determined using the ^1^H-NMR Nightingale platform for the quantification of 250 parameters of metabolism, including routine lipids, extensive lipoprotein subclasses, fatty acid composition, and various low-molecular metabolites, including amino acids, ketone bodies, and gluconeogenesis-related metabolites. Night shift workers had an increased BMI (26.6 vs. 25.9 kg/m^2^) compared with day workers (non-shift workers) in both sexes, were slightly more likely to be ever smokers (only in males) (54% vs. 46%), worked on average 5.9 ± 3.7 night shifts per month, and had been working in night shifts for 18.3 ± 10.5 years on average. We observed changes in several metabolic markers in male night shift workers compared with non-shift workers, but no changes were observed in women. In men, we observed higher levels of glycoprotein acetyls (GlycA), triglycerides, and fatty acids compared with non-shift workers. The changes were seen in the ratio of triglycerides and cholesterol(esters) to total lipids in different sizes of VLDL particles. Glycoprotein acetyls (GlycAs) are of particular interest as markers since they are known as biomarkers for low-grade chronic inflammation. When the analyses were adjusted for BMI, no significant associations were observed. Further studies are needed to better understand the relationship between night shift work and metabolic profiles, particularly with respect to the role of sex and BMI in this relationship.

## 1. Introduction

Surveys in Europe estimate that approximately 19% of the workers in the European Union (EU) work during the night at least once per month [1]. Over the past decades, night shift work has been associated with multiple adverse health effects. These include, for example, an increased risk of cardiovascular diseases, diabetes, cancer, and greater respiratory infection susceptibility [2,3,4,5,6,7]. Furthermore, night shift work has been associated with cardiovascular mortality, breast-cancer mortality, and all-cause mortality [7,8,9].

Working during the night disturbs the circadian rhythms of many physiological processes in the human body [10,11]. A widely supported hypothesis is that the desynchronization of the internal circadian rhythms underlies the health effects associated with night shift work. For example, it is proposed that eating during the night alters glucose metabolism [12,13], and a single night of sleep deprivation results in the alteration of metabolites [14]. Despite the great advancements in our understanding of the biological clock over the past decades, the precise mechanisms underlying the health effects of night shift work are still incompletely understood. Metabolomics assays in the blood have recently gained much attention as a minimally invasive biomarker platform. It has been shown that levels of metabolites in the blood are driven by environmental and behavioral time cues (e.g., eating or sleeping), and they appear to be predictive of disease onset, in particular for diseases associated with night shift work [15,16]. 

Previously, we showed that characteristics of night shift work are associated with several risk factors for metabolic diseases, such as BMI [17]. However, association patterns with metabolites such as cholesterol and glucose were much less clear [17]. Therefore, in the current study, we further explored the metabolic profiles of 1010 night shift workers and 1010 age- and sex-matched non-shift workers who participated in the Lifelines Cohort Study. The metabolic profiles consisted of 250 metabolites that were measured by the Nightingale platform, including routine lipids, extensive lipoprotein subclasses, fatty acid composition, and various low-molecular metabolites, including amino acids, ketone bodies, and gluconeogenesis-related metabolites [16]. 

## 2. Material and Methods

### 2.1. Study Population and Design

We used data from participants in the Lifelines Cohort Study. Lifelines is a multi-disciplinary prospective population-based cohort study with a unique three-generation design examining the health and health-related behaviors of 167,729 persons living in the north of the Netherlands. It employs a broad range of investigative procedures in assessing the biomedical, socio-demographic, behavioral, physical, and psychological factors that contribute to the health and diseases of the general population, with a special focus on multi-morbidity and complex genetics. The overall design and details of the methodology of the Lifelines cohort study can be found in previous papers [18,19,20]. 

Participants of the Lifelines Cohort study were recruited between 2007–2013 and were requested to visit one of the 12 Lifelines research facilities for a basic medical examination every 5 years. For the purpose of the current study, participants in the Lifelines cohort aged ≥18 years with valid email addresses (*n* = 78,190) were approached in December 2017 and January 2018 for an additional questionnaire about their shift work history. Questions involved the participants’ shift work histories (including early morning, evening, and night shifts) and work schedules during the three months before blood samples were drawn in the second assessment round (2014–2017). The Lifelines study has been approved by the Medical Ethics Committee of the University Medical Centre Groningen, the Netherlands (under number 2007/152). Written informed consent was obtained from all participants before entering the study [19].

### 2.2. Data and Sample Collection

**Figure 1:**
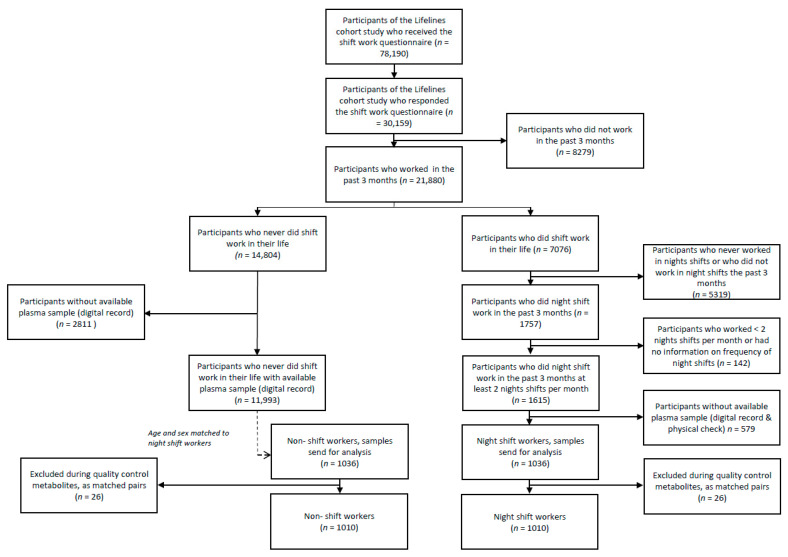
Flowchart of inclusion and exclusion of study population.

In total, 30,159 individuals (38.6%) responded to the shift work questionnaire. The questionnaire on shift work was designed to cover the major characteristics of shift work: current shift work status, frequency of night shifts (on average per month), and duration of night shift work in years [21,22]. Workers (>12 h per week) were defined as night shift workers if they worked at least 3 h between 00.00 and 05.00 [21], either in a rotating or permanent schedule. Non-shift workers were participants who never performed shift work (i.e., no early morning, evening, or night shifts) and had regular work times during the three months prior to the blood draw (starting work after 06.00 and finishing work before 21.00). Participants were only included if a plasma sample was available, and night shift workers were included if they worked at least 2 nights per month during the three months before the blood draw. This resulted in the inclusion of 1036 night shift workers. Each night shift worker was randomly matched on the basis of sex and age with a non-shift worker (*n* = 1036) from the group of 11,993 non-shift workers (Figure 1).

As requested, 98% of the participants fasted overnight before the blood draw between 08.00 and 10.00 at one of the Lifelines research facilities (self-reported). Plasma samples were stored by Lifelines Biobank at −80 °C. 

### 2.3. Covariates

Data on age (in years), sex (male/female), smoking (current/former/never), and BMI (kg/m^2^) were obtained from the Lifelines Cohort database (second assessment round (2014–2017)). These covariates were included because they are known to be associated with night shift work and/or metabolite levels [17,23,24,25,26,27]. Age, sex, and smoking were self-reported. BMI was calculated on the basis of weight and height measurements during the second assessment round. Weight and height measurements were taken in light clothing without shoes. Body weight was measured to the nearest 0.1 kg. Height was measured to the nearest 0.5 cm.

### 2.4. Metabolomics Measurements

The 2072 included samples were sent to Nightingale Health Plc for high-throughput NMR spectroscopy in 2020. This analysis provided simultaneous quantification of 250 metabolic biomarkers measures in a single assay and included lipids, lipoproteins, fatty acid composition, various low-molecular-weight amino acids, and their derived measures (e.g., ratios). For more details see Blood Biomarker Analysis (nightingalehealth.com) (accessed on 18 November 2022). Measurements of the metabolic markers were conducted blinded. 

### 2.5. Statistical Methods

Preprocessing: Following Nightingale Health indications, we excluded 20 contaminated samples (18 tagged with “high ethanol” and 2 with “high lactate”), together with their respective matched sample (day/night worker). We also decided to exclude 12 samples (6 day and 6 night workers) with >5 missing or zero values for metabolite levels. The remaining 419 missing values (0.083% of the dataset) were imputed using the function nipals of the R package pcaMethods [28]. Finally, the metabolic features were normalized using z-scaling after applying a natural logarithmic transformation (see Supplementary Figure S6 for their distributions). To avoid problems with log transformation, a value of 1 was added to all biomarkers containing zeroes (glycine and beta-hydroxybutyrate), indicating a value below the detection limit, as previously carriedout by other studies [29,30]. Principal Component Analysis (PCA): As a preliminary analysis to identify the sources of greatest variance in the metabolomics dataset, we performed the PCA (with the function prcomp in R). To evaluate which covariates had the greatest influence on the metabolomics dataset, we studied the correlation between the first 15 principal components (~90% of the variance) and the covariates. Covariates: Differences in age, smoking, BMI, and night shift work characteristics between night shift workers and non-shift workers were tested with the Mann-Whitney U test, t-test, or Pearson Chi-square test. Metabolome-wide association studies (MetaboWAS): Linear regression models on the metabolomics quantifications, adjusted for potential confounders (based on the results of the previous section), were used to study the associations between each metabolite and night shift work status. The false discovery rate (FDR) was applied to account for multiple testing. Multi-biomarker scores: Recently, several multi-biomarker scores based on metabolomics data have been developed that are predictive of long-term health outcomes [29,31]. The coefficients indicated by Deelen et al. were used to obtain the MetaboHealth score, whose name was given later to the score in the Deelen et al. paper. We also calculated the [29] MetaboAge scores, as previously described den Akker et al., with an updated version that takes into account the changes in the Nightingale platform, which were made in 2020 [31]. The MetaboAge score was trained on a large set of cohorts with Nightingale Metabolomics data on the basis of chronological age; the MetaboHealth score was also trained on a large set of cohorts on the basis of mortality data. Both scores were constructed to generate indicators of biological age. We used t-tests to compare the MetaboHealth score and MetabAge score between night shift workers and non-shift workers in men and women separately.

## 3. Results

### 3.1. Description of the Study Population and Dataset

The final dataset after quality control (see methods section) consisted of 938 women (*n* = 469 non-shift workers and *n* = 469 night shift workers) and 1082 men (*n* = 541 non-shift workers and *n* = 541 night shift workers). Women were, on average, 45.0 years old, and men were, on average, 47.7 years old (Table 1). In both women and men, night shift workers had an increased BMI (Table 1). Among men, slightly more non-shift workers were never smokers (54%) compared with night shift workers (46%). Night shift workers worked an average of 5.9 ± 3.7 night shifts per month, with men working slightly more night shifts per month on average compared with women (6.8 ± 3.8 and 5.9 ± 3.7, respectively; *p* < 0.001). Night shift workers had been working night shifts for 18.3 years on average, with men working night shifts slightly longer than women (18.9 ± 10.7 vs. 17.6 ± 10.1 years; *p* = 0.046). There were no differences in the percentages of participants with diabetes mellitus type 2, hypertension, or high cholesterol in any of the groups (Supplemental Table S1). 

### 3.2. Associations between Metabolite Markers and Night Shift Work

**Figure 2:**
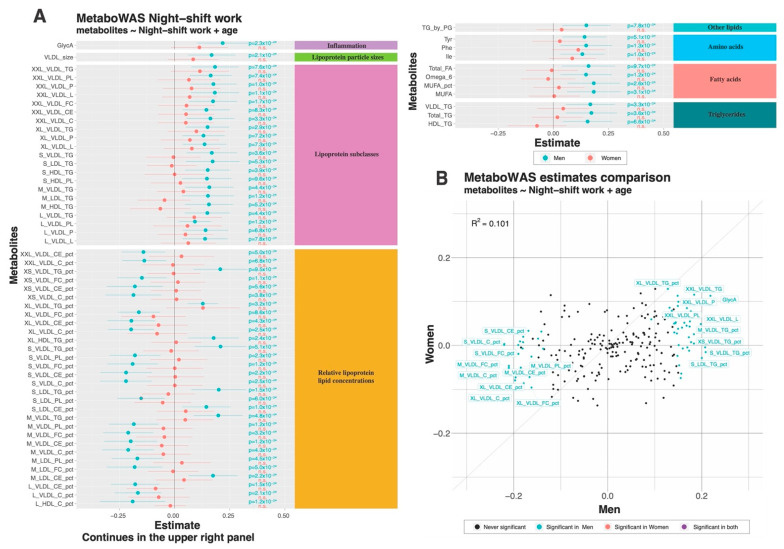
Univariate associations between metabolites (or metabolite ratios) and night shift work, significant in men. (**A**): In men (blue), positive associations were observed between several types of metabolites ranging from inflammation to triglycerides. Negative associations were observed in relative lipoprotein concentrations. In women (pink), no significant associations were observed after Benjamini Hochberg correction. * indicates significant associations and n.s stands for non significant. (**B**): Scatterplot comparing the effect estimates in all metabolites in the Nightingale platform in men (on the *x*-axis) and women (on the *y*-axis). Highlighted in blue and tagged are the metabolites significantly associated in men. No metabolite was associated with night shift work in women; therefore, no points are colored red or purple. The analyses are adjusted for age.

A principal component analysis (PCA) showed that most of the variance in the metabolomics dataset could be attributed to sex differences (Supplementary Figure S1). While a clear separation was visible for sex (Supplemental Figure S1D), no clear separation could be made on the basis of night shift work exposure (Supplemental Figure S1C). Concomitantly, association analyses were conducted using sex-stratified analyses. For the results of the non-stratified analyses, see Supplementary Figure S5. Linear regression analyses adjusted for age showed that in male night shift workers, 64 out of 250 metabolites were significantly associated with night shift work (either positively or negatively, Figure 2A). By contrast, none of the 250 metabolites were significantly associated with night shift work in women. Figure 2B shows the effect estimates of all metabolite markers significantly associated with night shift work and visualizes these different results for men and women. 

In men, positive associations were observed in eight different subgroups of metabolites (as determined by Nightingale Health), ranging from an inflammation marker to triglycerides. Negative associations were only observed in a single group, yet this was the largest group: ‘relative lipoprotein lipid concentrations’ (Figure 2A). The markers positively associated with the largest effect sizes in male night shift workers included ‘glycoprotein acetyls’ (GlycA, estimate~0.219, *p*~2.3 × 10^−4^) and ‘triglycerides to total lipids ratio in small VLDL’ (S_VLDL_TG_pct, estimate~0.208, *p*~5.1 × 10^−4^). The largest negative effect sizes in male night shift workers included ‘cholesteryl esters to total lipids ratio in small VLDL’ (S_VLDL_CE_pct, estimate~−0.22, *p*~2.2 × 10^−4^) and ‘cholesterol to total lipids ratio in small VLDL’ (S_VLDL_C_pct, estimate~−0.219, *p*~2.5 × 10^−4^) (Figure 2 and Figure S2). 

**Figure 3:**
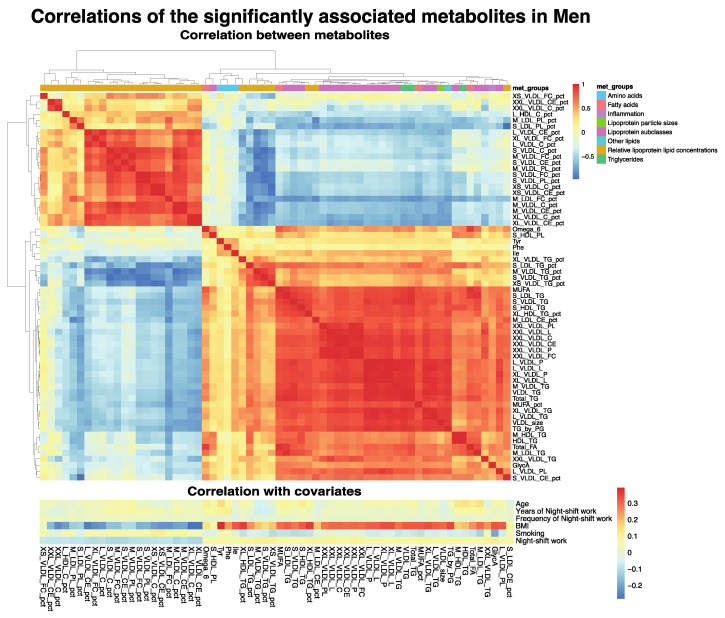
Correlations of the metabolite markers in males that were significantly associated with night shift work, including correlation with covariates.

The group of ‘lipoprotein subclasses’ and ‘relative lipoprotein lipid concentrations’ included a large set of makers related to different sizes of VLDL particles. In particular, markers indicative of the ratio of triglycerides and cholesterol(esters) to total lipids in different sizes of VLDL particles were associated with night shift work in men. The total levels of triglycerides (Total_TG, estimate~0.17, *p*~3.6 × 10^−3^) and fatty acids (Total_FA, estimate~0.16, *p*~9.7 × 10^−3^) were also significantly increased in male night shift workers (Figure 2A). The pairwise correlation analyses of all significant markers in men indicated several subgroups of markers with higher positive or negative correlations (Figure 3). The markers within the group of ‘relative lipoprotein lipid concentration’ clustered together, which was not surprising considering that these were strongly related markers. Similar patterns of correlations were also observed with several of the covariates, with BMI being the most relevant in the male dataset (Figure 3), which suggests that the observed associations between metabolite levels and night shift work in males are related to differences in BMI. Indeed, when BMI was included as a confounder in the univariate linear regressions, none of the metabolite markers were significantly associated with night shift work (Supplemental Figure S2). When smoking was included in the model, ~50% of the metabolite markers that were significantly associated in the crude model no longer reach the significance threshold (Supplemental Figure S2). 

### 3.3. Multi-Biomarker Scores

Recently, several multi-biomarker scores based on metabolomics data have been developed to generate indicators of biological age and are predictive of long-term cardio-metabolic health outcomes [29,31]. Some of the markers associating with night shift work (in men), such as GlycA, are part of these multi-biomarker scores. However, in our study, no association of night shift work with the MetaboHealth score or MetaboAge was observed (Supplemental Figure S3). 

### 3.4. Characteristics of Night Shift Work

**Figure 4:**
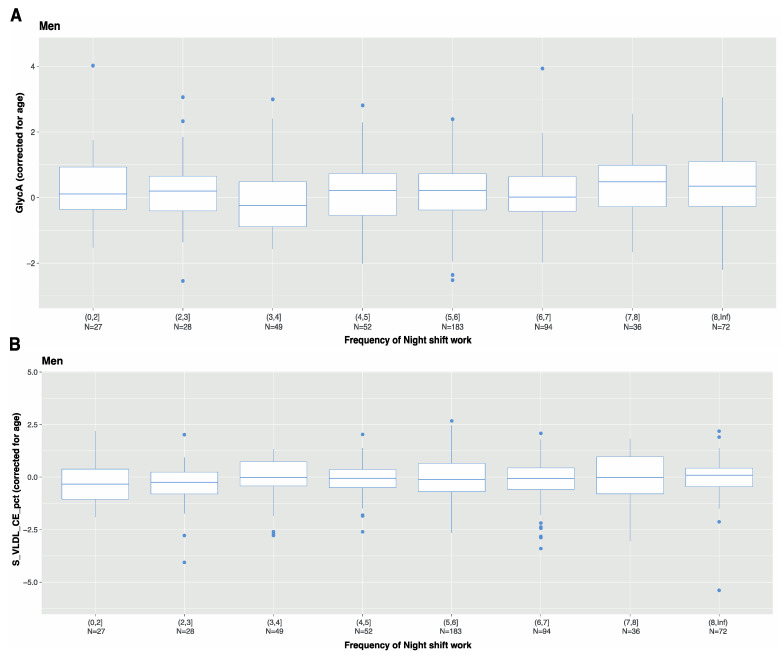
Residuals of (**A**) GlycA and (**B**) S_VLDL_CE_pct after correcting for age among male night shift workers based on average frequency of night shift per month: ≤2 night shifts per month, >2–≤3 nights shifts per month, >3–≤4 nights shifts per month, >4–≤5 nights shifts per month, >5–≤6 nights shifts per month, >6–≤7 nights shifts per month, >7–≤8 nights shifts per month, and >8 nights shifts per month.

**Figure 5:**
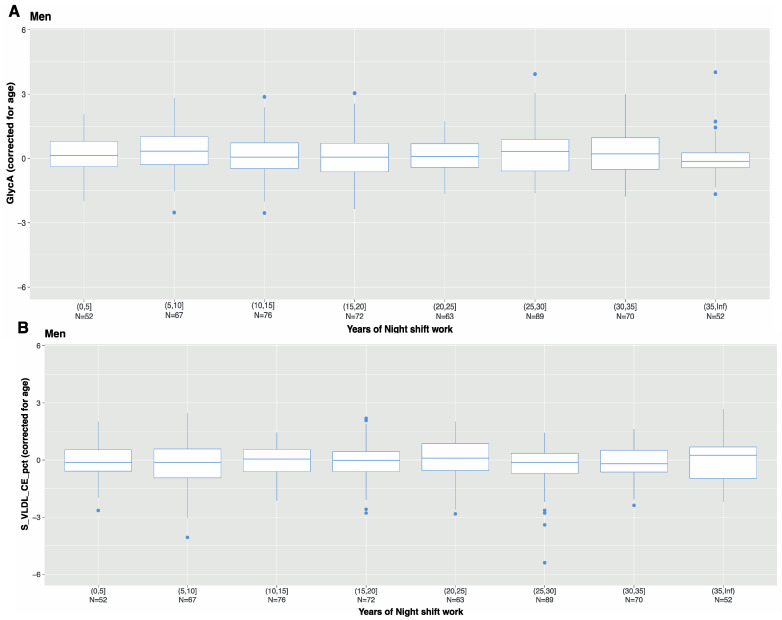
Residuals of (**A**) GlycA and (**B**) S_VLDL_CE_pct, after correcting for age among male night shift workers based on years of night shift: ≤5 years of night shifts, >5–≤10 years of nights shifts per month, >10–≤15 years of nights shifts, >15–≤20 years of nights shifts, >20–≤25 years of nights shifts, >25–≤30 years of nights shifts, >30–≤35 years of nights shifts, and >35 years of nights shifts.

Previously, we showed that characteristics of night shift work, such as frequency and duration, were associated with metabolic risk factors, such as BMI [17]. Therefore, we investigated the levels of GlycA and S_VLDL_CE_pct among night shift workers on the basis of frequency (average number of night shifts per month) and duration (number of years engaged in night shift work). GlycA and S_VLDL_CE_pct were the two markers with the largest effect estimates for a positive and negative association, respectively. Furthermore, they might represent different signals, as they are not highly correlated positively or negatively (Figure 3). Nevertheless, we observed no differences in GlyCA or S_VLDL_CE levels among male night shift workers related to the frequency of night shift work (Figure 4) or the duration of night shift work (Figure 5). 

## 4. Discussion

In the current cross-sectional study, we explored the metabolic profiles of 1010 night shift workers and 1010 age- and sex-matched non-shift workers from the Lifelines Cohort Study. Both sexes had differences in BMI associated with night work, and we observed changes in several metabolic markers in male night shift workers but not in women. In men, we observed an increase in the levels of glycoprotein acetyls (GlycA), triglycerides, and fatty acids. The changes in triglycerides were mainly related to the ratio of triglycerides and cholesterol(esters) to total lipids in different sizes of VLDL particles. Since correction for BMI decreased the effect in men, we conclude that the increased BMI associated with night work coincides with differences in metabolomic parameters only in men, especially as an indicator of low-grade inflammation (Glyc A).

To our knowledge, this is the first time GlycA has been investigated in night shift workers. GlycA is a nuclear magnetic resonance (NMR)-derived signal that originates from a subset of glycan *N*-acetylglucosamine residues on enzymatically glycosylated acute-phase proteins (predominantly alpha−1-acid glycoprotein, haptoglobin, and alpha-1-antitrypsin) [32,33]. In the past few years, studies have investigated GlycA as a biomarker for chronic systemic inflammation. Higher levels of GlycA have been linked to both type 2 diabetes and cardiovascular disease risks in several cohort studies [33,34,35,36] as well as to a higher risk of severe infections [37]. Considering the known association of night shift work with diabetes type 2, cardiovascular diseases, and infection susceptibility [6], our finding that GlycA was increased in male night shift workers is of particular interest. 

In addition to changes in GlycA, we observed increases in several metabolic markers related to the ratio of triglycerides and cholesterol(esters) to total lipids in different sizes of VLDL particles as well as an increase in total triglycerides. These results are in line with those of a recent large meta-analysis: ‘night shift work was associated with increased levels of triglycerides based on 48 previously published studies’. Importantly, no differences between men and women were reported in this meta-analysis, as the authors noted that women were generally underrepresented in the included studies, and therefore, sex-stratified analyses could not be performed [38]. By contrast, the current study was sex-balanced and able to recover the previously reported association between night shift work and increased levels of triglycerides, yet only in men. This suggests that biomarker risk profiles trained in men might be of limited utility when applied to females, as is the case with more established cardio-metabolic risk factors. It also potentially points to a difference in the molecular mechanisms that underly the adverse health associations of night shift work in males and females.

Our study indicated differences in the metabolic profiles of men and women, with no significant differences between night-shift workers and non-shift workers observed in women. When we applied sex stratification to our previously published dataset [17], we also observed sex differences. For example, triglycerides and the number of leukocytes were increased only in male night shift workers compared with non-shift workers (data not published). Previous studies have shown that one-third of 507 metabolic markers differed between men and women [23,25] and that age- and diet-related changes in the metabolome differed between men and women [23,39]. Furthermore, it is known that the link between obesity and the risk of cardiovascular disease differs between men and women {Palmer, 2015 #64}. It has been hypothesized that, for example, differences in fat distribution could underlie the sexual dimorphism observed in cardiovascular disease risk factors. In premenopausal women, fat accumulates more in subcutaneous depots, while men tend to have more visceral fat. This distribution changes in women after menopause and is linked to hormonal changes {Palmer, 2015 #64}. Levels of metabolites in women are also influenced by their menstrual cycles [23]. Unfortunately, in our study, the phase of the menstrual cycle or menopause stage at the time of the blood draw was unknown and could not be taken into account. It could be that the relationship between night shift work, metabolites, and BMI changes in women after menopause and explains the differences observed in our study between men and women. These results emphasize the importance of including sex-stratified analysis in future studies, as well as information regarding the menstrual cycle and menopause stages in women. 

To our knowledge, this is one of the first studies investigating blood metabolomic profiles with 250 metabolic markers in night shift workers. We are aware of one previous study in which metabolomic analysis was performed in a small group of female night shift workers (*n* = 15) and age- and BMI-matched female non-shift workers (*n* = 15) [40]. The authors reported 76 metabolites that were significantly changed in night shift workers, including L-tryptophan, acylcarnitines, and several fatty acids. One other study investigated metabolites in night shift workers but performed analyses on urine samples [41]. One marker was consistently changed in this study: acylcarnitine C10:2. Due to the lack of standardization and the difference in coverage, it is difficult to compare metabolite datasets across studies using different detection methods [42]. Therefore, it is difficult to directly compare the results of these two studies to our own.

In our study, BMI was increased in both male and female night shift workers, and levels of GlycA were correlated with BMI in both males (Figure 3) and females (Supplemental Figure S4). However, a significant association of night shift work with increased GlycA was only observed in males. It is hypothesized that GlycA, a marker for chronic inflammation, may coincide with obesity, and weight loss after bariatric surgery is related to a decrease in GlycA levels [43]. However, GlycA also predicts cardiovascular disease risks irrespective of BMI [35]. As GlycA was relatively recently identified as a biomarker; the causality of relationships is still largely unknown. Increases in GlycA could, for example, be secondary to changes in BMI or related to different eating patterns in night shift workers. For example, a study in healthy individuals showed large variability in the daytime postprandial response of GlycA {Mazidi, 2021 #65}. Previous studies have shown that a reduction in elevated Glyc A is feasible by different interventions, such as exercise, bariatric surgery, and dietary interventions, including gut microbiome changes {Barber, 2018 #67;Manmadhan, 2019 #40;Vijay, 2021 #68}. Most of these interventions also resulted in weight loss, replicating the association with body weight (BMI) we found in our analysis. Nevertheless, the intervention studies suggest GlycA can be reduced in night shift workers as well. Further studies are needed to gain more insight into the complex relationship between night shift work, BMI, sex differences, and changes in GlycA. 

In our study, we observed no association of frequency and duration of night shift work with the top two markers in men (GlycA and S_VLDL_CE). This is somewhat in line with our previous study, which indicated that characteristics of night shift work (such as the number of night shifts per month) are not clearly associated with metabolites, such as cholesterol and glucose [17]. One explanation previously suggested for the absence of a dose-response effect in night-shift workers is the healthy worker effect [44,45,46]. This refers to the notion that workers who are able to work many night shifts/month or for more years represent a relatively healthy group. In addition, a disadvantage of the frequency and duration measures used in the current study is that they do not take into account other important aspects of the shift schedule, such as the number of consecutive night shifts and the number of rotations between day and night shifts. These are known to also be important aspects of night-shift work [22]. For example, a previous meta-analysis indicated that permanent night shifts were associated with dyslipidemia, mainly via elevated total cholesterol and triglycerides, and reduced HDL-cholesterol in night shift workers [38]. 

One important limitation of our current study is that we do not have information on whether night shift workers worked night shifts during the days directly prior to the blood draw. For instance, previous studies have shown that the rhythmicity of metabolites is altered during or directly after simulated night shifts [24]. Kervezee et al. showed that in nine healthy subjects (eight men and one woman), four days of simulated night shifts affect the rhythmicity and acrophase of several metabolites [47]. It was also noted that the response to the simulated night shifts was highly diverse among individuals, with phase shifts of rhythmic metabolite profiles ranging from a 0.2-h advance in one subject to a 12-h delay in another subject [47]. While population studies, including ours, generally cannot collect data in such great detail as these rhythmicity studies, they do have the advantage of considerably larger sample sizes that allow for the robust identification of night shift work being associated with metabolites with smaller effect sizes. Further studies could include a question on working night shifts during the week prior to the blood draw to further take into account any effect of night shifts on the diurnal patterns of metabolites. 

To summarize, we observed changes in several metabolic markers in male night shift workers but not in women, despite the increased BMI in both female and male night shift workers. In male night shift workers, we observed that BMI was associated with higher levels of GlycA, triglycerides, and fatty acids markers compared with non-night shift workers. Further studies are needed to better understand the relationship between night shift work and these metabolic markers, with respect to the role of sex, BMI, and characteristics of night shift work in this relationship. 

## Figures and Tables

**Table 1:** Characteristics of the study population.

Study Population (*n* = 2020)	Non-Shift Workers (*n* = 1010)	Night Shift Workers (*n* = 1010)
Age (in years, mean (SD))	46.4 (8.5)	46.4 (8.5)
Sex (% male)	53.6	53.6
BMI (in kg/M^2^, mean (SD))	25.9 (4.0)	26.6 (4.4) *
Smoking (current/former/never, %) (*n* = 1922)		
Frequency of night shifts/month (mean, SD)	n.a.	5.9 (3.7)
Duration of night shifts in years (mean, SD)	n.a.	18.3 (10.5)
**Men (*n* = 1082)**	**Non-shift workers (*n* = 541)**	**Night shift workers (*n* = 541)**
Age (in years, mean (SD))	47.7 (8.1)	47.7 (8.1)
BMI (in kg/M^2^, mean (SD))	26.1 (3.6)	26.9 (3.9) *
Smoking (current/former/never, %) (*n* = 1030)	19/27/54%	21/34/46% *
Frequency of night shifts/ month (mean, SD)	n.a.	6.8 (3.8)
Duration of night shifts in years (mean, SD)	n.a.	18.9 (10.7)
**Women (*n* = 938)**	**Non-shift workers (*n* = 469)**	**Night shift workers (*n* = 469)**
Age (in years, mean (SD))	45.0 (8.8)	45.0 (8.8)
BMI (in kg/M^2^, mean (SD))	25.5 (4.4)	26.2 (4.8) *
Smoking (current/former/never, %) (*n* = 892)	11/28/61%	15/32/55%
Frequency of night shifts/ month (mean, SD)	n.a.	4.8 (3.1)
Duration of night shifts in years (mean, SD)	n.a.	17.6 (10.1)

* *p* < 0.05, Mann-Whitney U test or Pearson Chi-square test. SD = standard deviation; n.a. = not applicable.

## Data Availability

Third Party Data: Restrictions apply to the availability of these data. Data used in this study were obtained from the Lifelines Cohort Study and can be requested via www.lifelines.nl/researcher (accessed from 1 June 2021 until 1 November 2022). Data are available upon request according to their permissions for use. Please see www.lifelines.nl/researcher (accessed on 1 June 2021).

## Supplementary Materials

The following supporting information can be downloaded at: https://www.mdpi.com/article/10.3390/metabo12121172/s1, Figure S1: Principal component analysis (PCA) of metabolite markers; Figure S2: Volcano plots of the association between individual metabolites and night shift work in males; Figure S3: Boxplot of mean MetaboHealth score and ΔMetaboAge score; Figure S4: Correlations of the metabolite markers, significantly associated with night shift work in men, with covariates in women; Figure S5: Univariate associations between metabolites (or metabolites ratios) and night shift work, significant in men; Figure S6: Histograms of the metabolomics features after log transformation and z-scaling; and Table S1: Disease information of the study population including stratification for sex.
