# Supplementary material for: GlycA, a Biomarker of Low-Grade Inflammation, Is Increased in Male Night Shift Workers
Source: Metabolites. 2022 Nov 24;12(12):1172. doi: 10.3390/metabo12121172 (PMC9785707; doi:10.3390/metabo12121172)
Supplement: Supplementary file 1 [file metabolites-12-01172-s001.zip › metabolites-2025837-supplementary.pdf]

Supplemental Figures

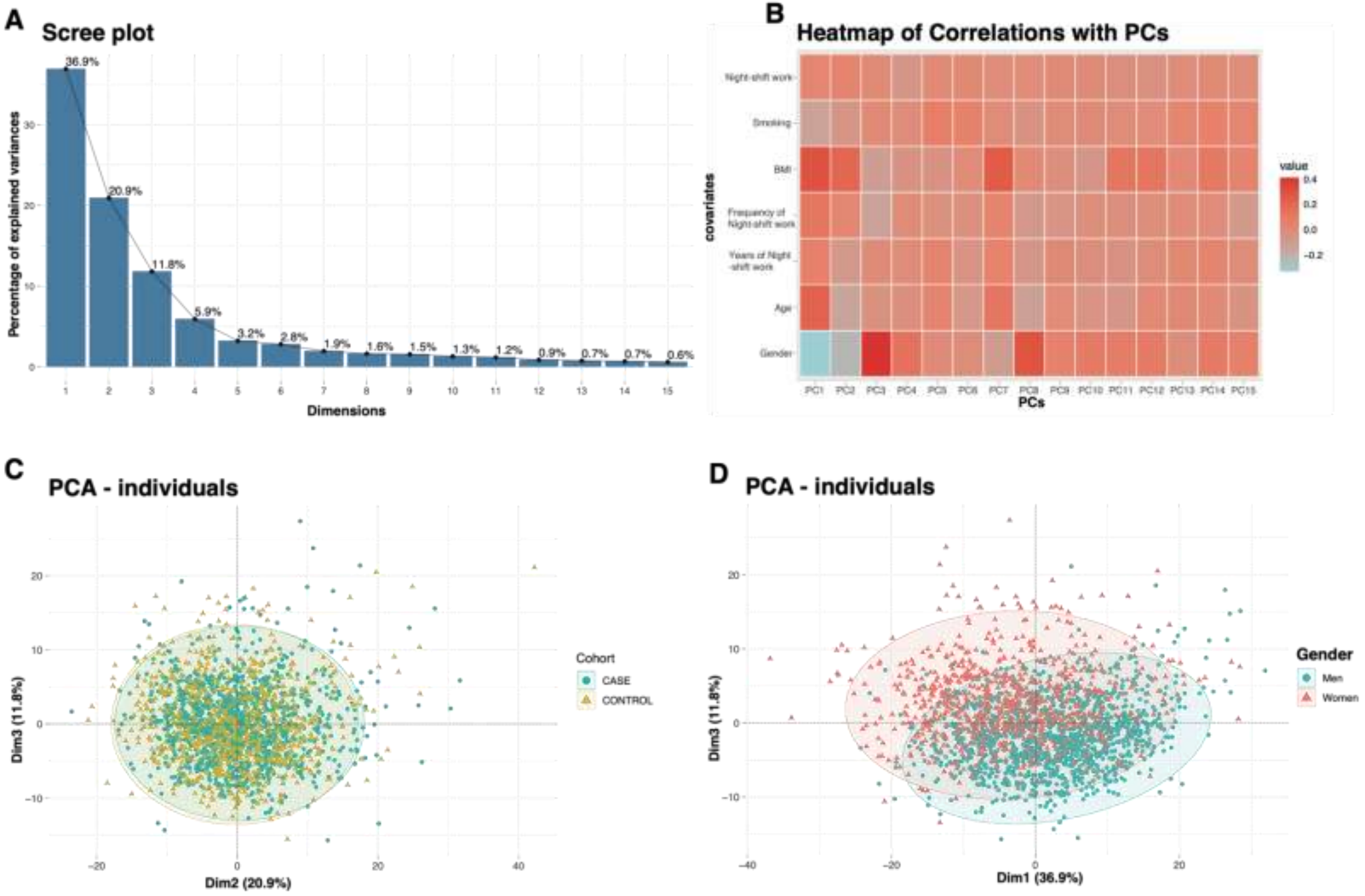

Supplemental Figure S1. Principal component analysis (PCA) of metabolite markers. **A:** Percentage of variance explained by each dimension. **B**: Heatmap of correlation of the first 15 dimensions with night shift work and covariates. **C**: PCA plot of dimensions 2 and 3 with visual contrast for night shift workers (blue dots) and non-shift workers (yellow triangles). **D:** PCA plot of dimension 1 and 3 with visual contrast for men (blue dots) and women (pink triangles).

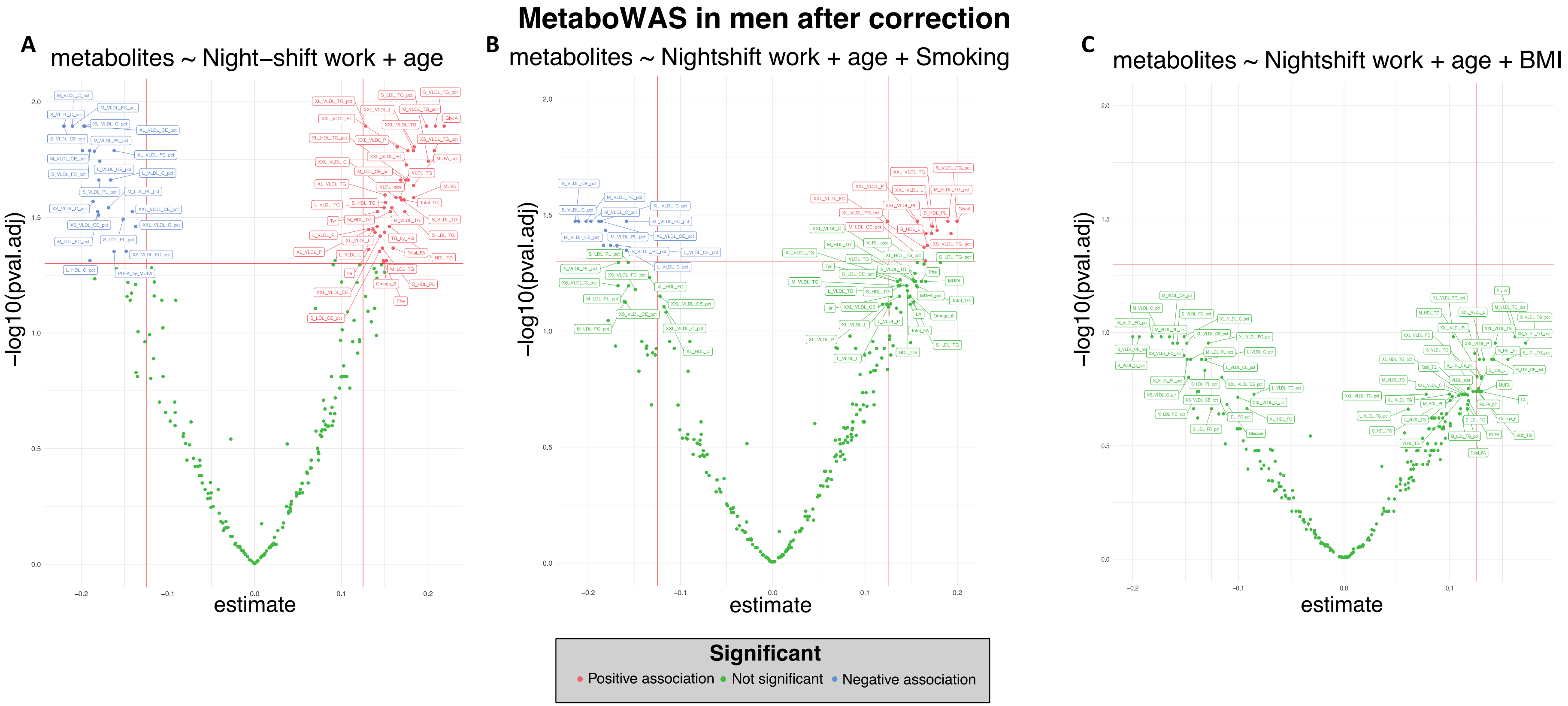

Supplemental figure S2 Volcano plots of the association between individual metabolites and night shift work in males including age **(A)**, age and smoking **(B)**, and age and BMI **(C)**. Positive associations are labeled red, negative associations are labeled blue, and non-significant associations are labeled green.

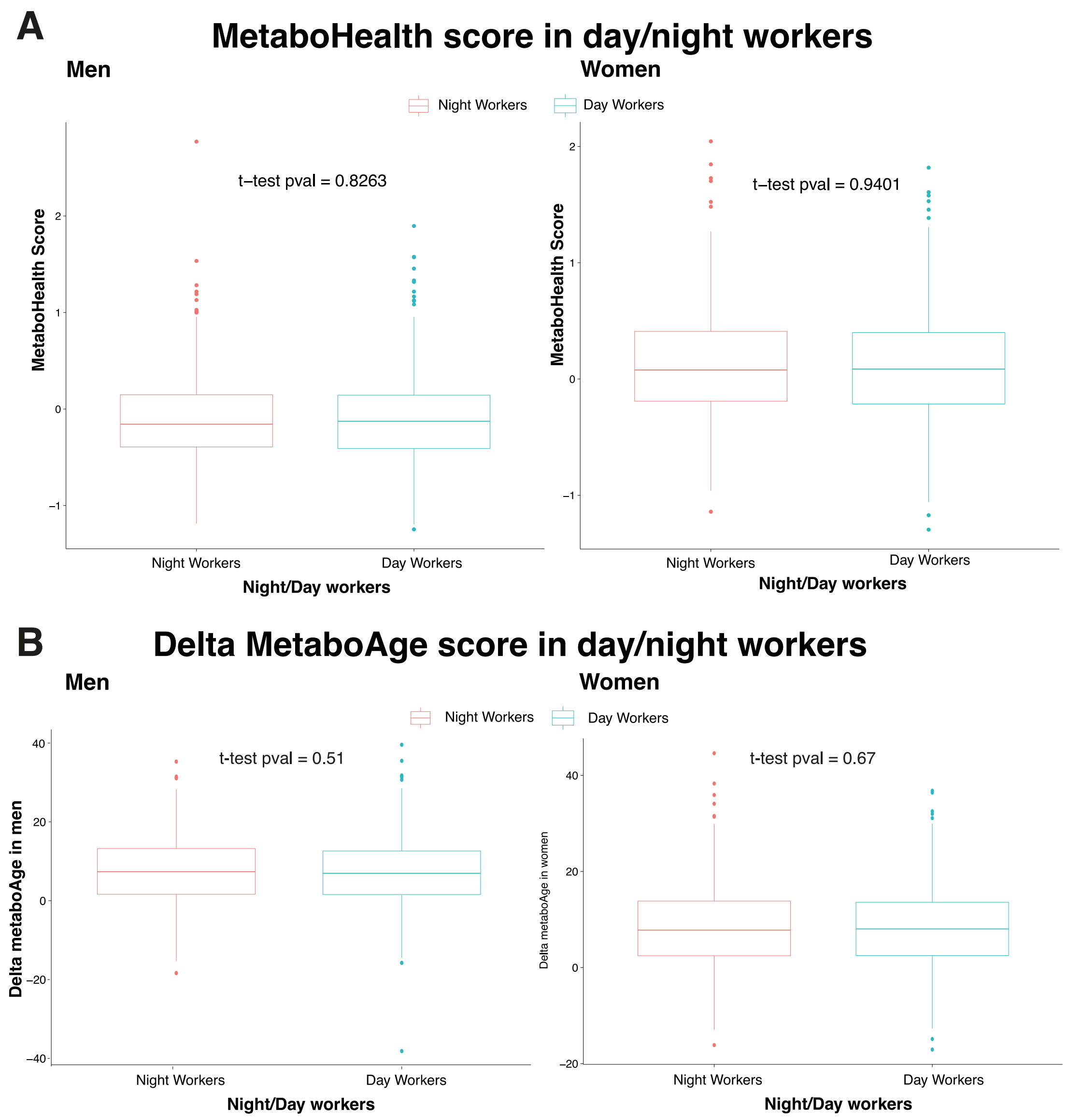

Supplemental Figure S3 Boxplot of mean MetaboHealth score **(A)** and ΔMetaboAge score **(B)** in night shift workers (red) and non-shift workers (blue) in men and women.

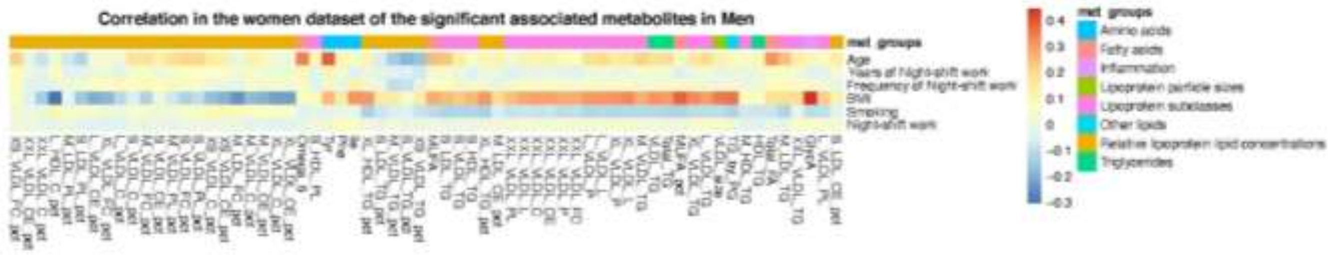

Supplemental Figure S4 Correlations of the metabolite markers, significantly associated with night shift work in men, with covariates in women.

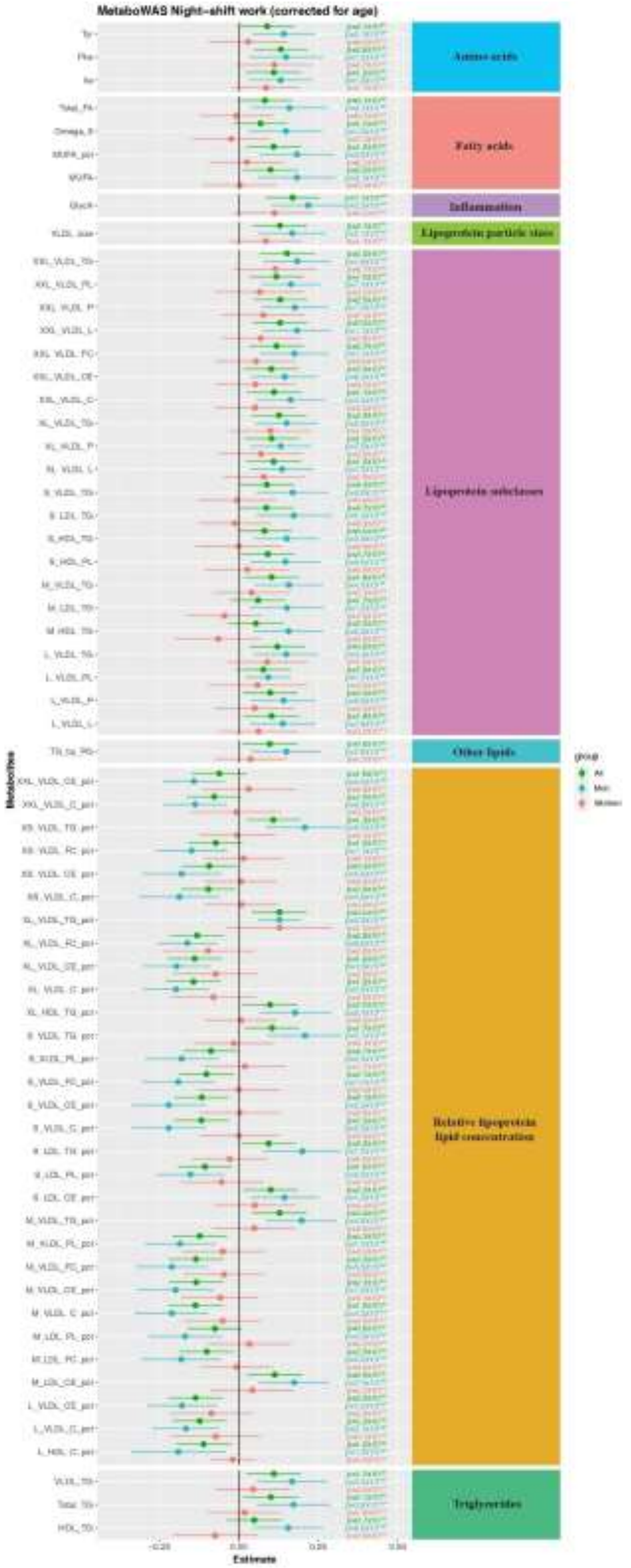

Supplemental Figure S5 Univariate associations between metabolites (or metabolites ratios) and night shift work, significant in men. In addition to the results shown in Figure 2, this present also the results of the non-stratified analyses, indicated as “All” (in green). The metabolites present no significant association in the non-stratified analysis after Benjamini Hochberg correction. * indicates significant associations.

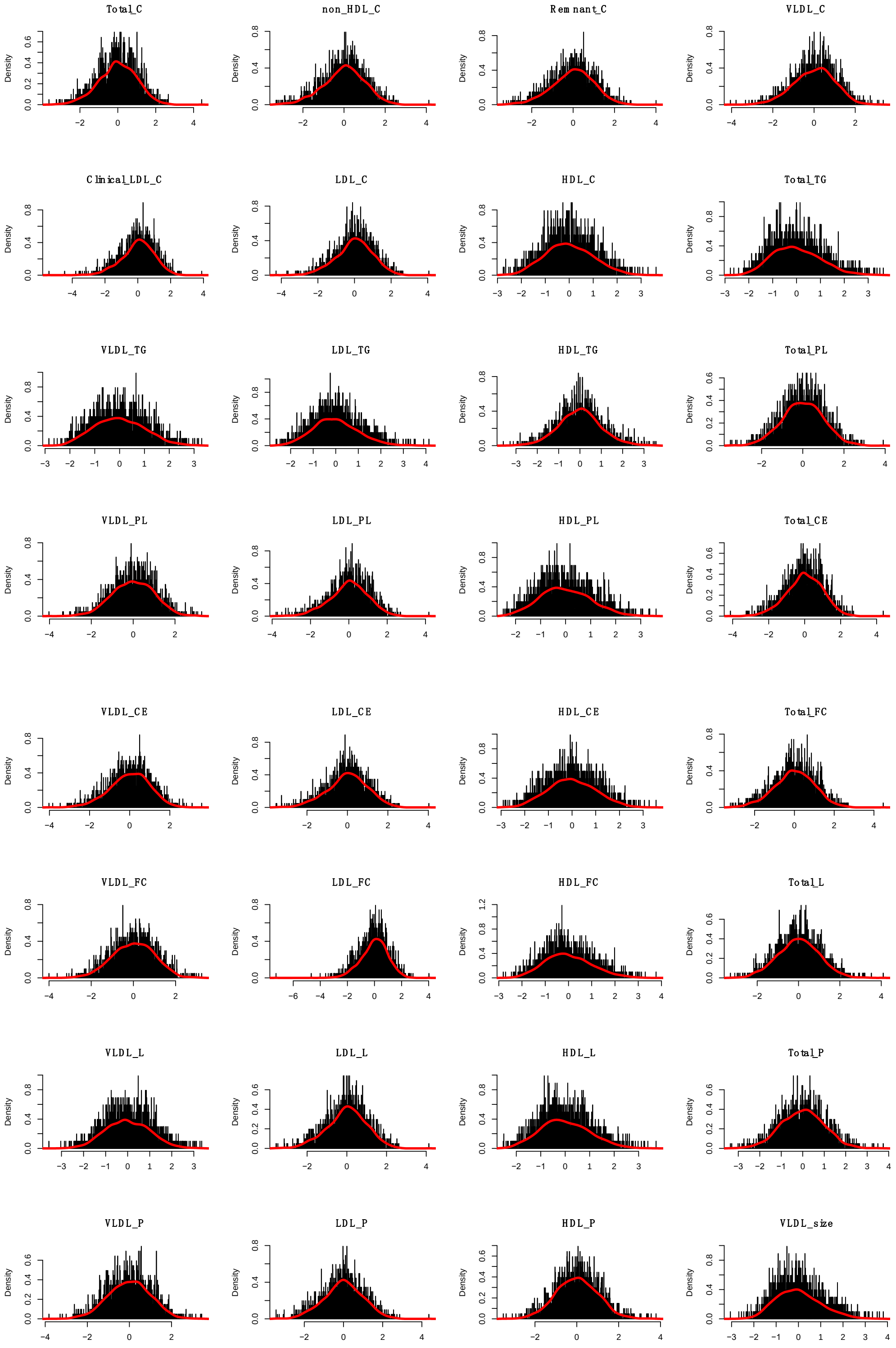

Supplemental Figure S6 Histograms of the metabolomics features after log transformation and z-scaling. Continues on the next page.

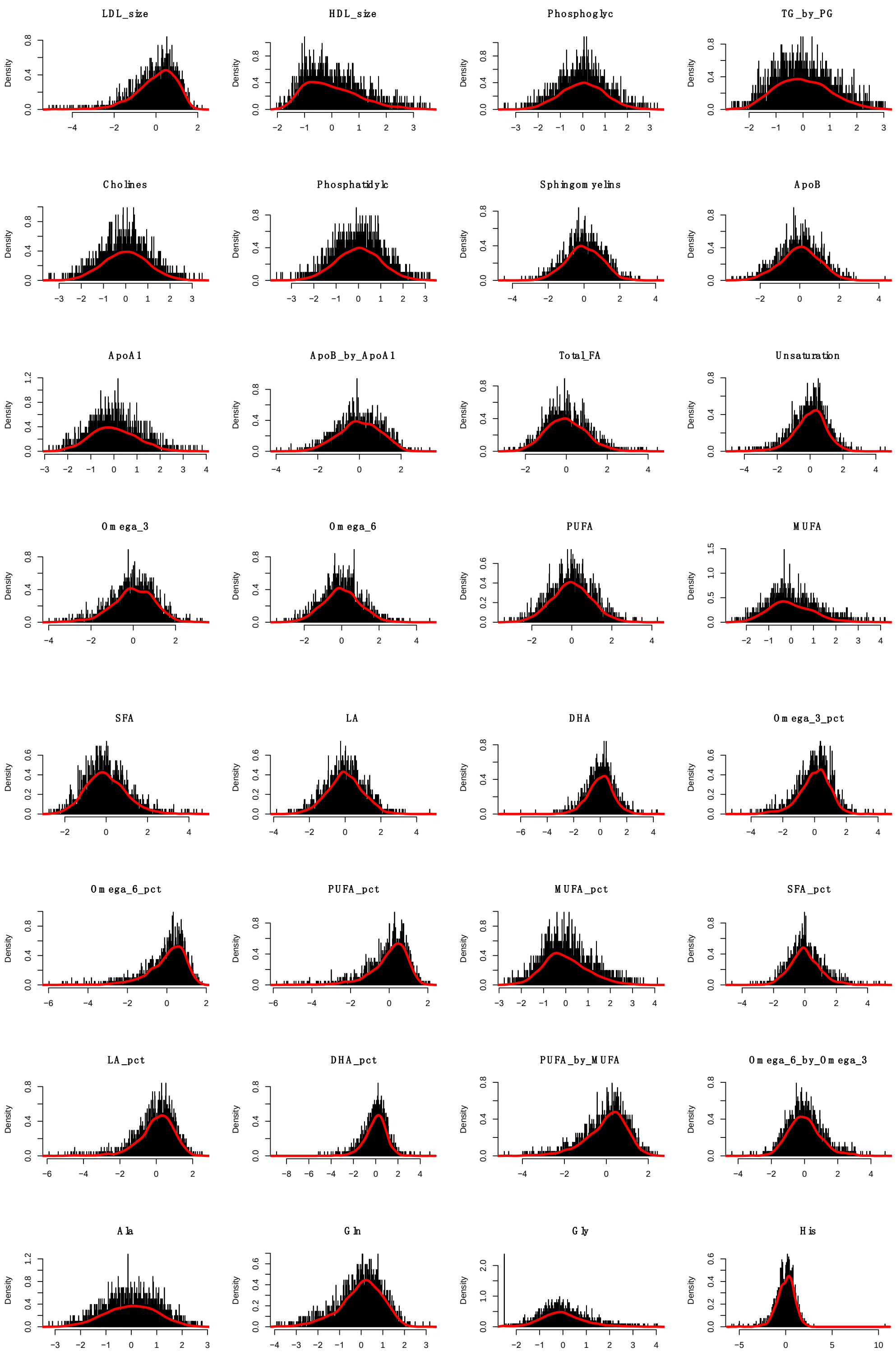

Supplemental Figure S6 Histograms of the metabolomics features after log transformation and z-scaling. Continues on the next page.

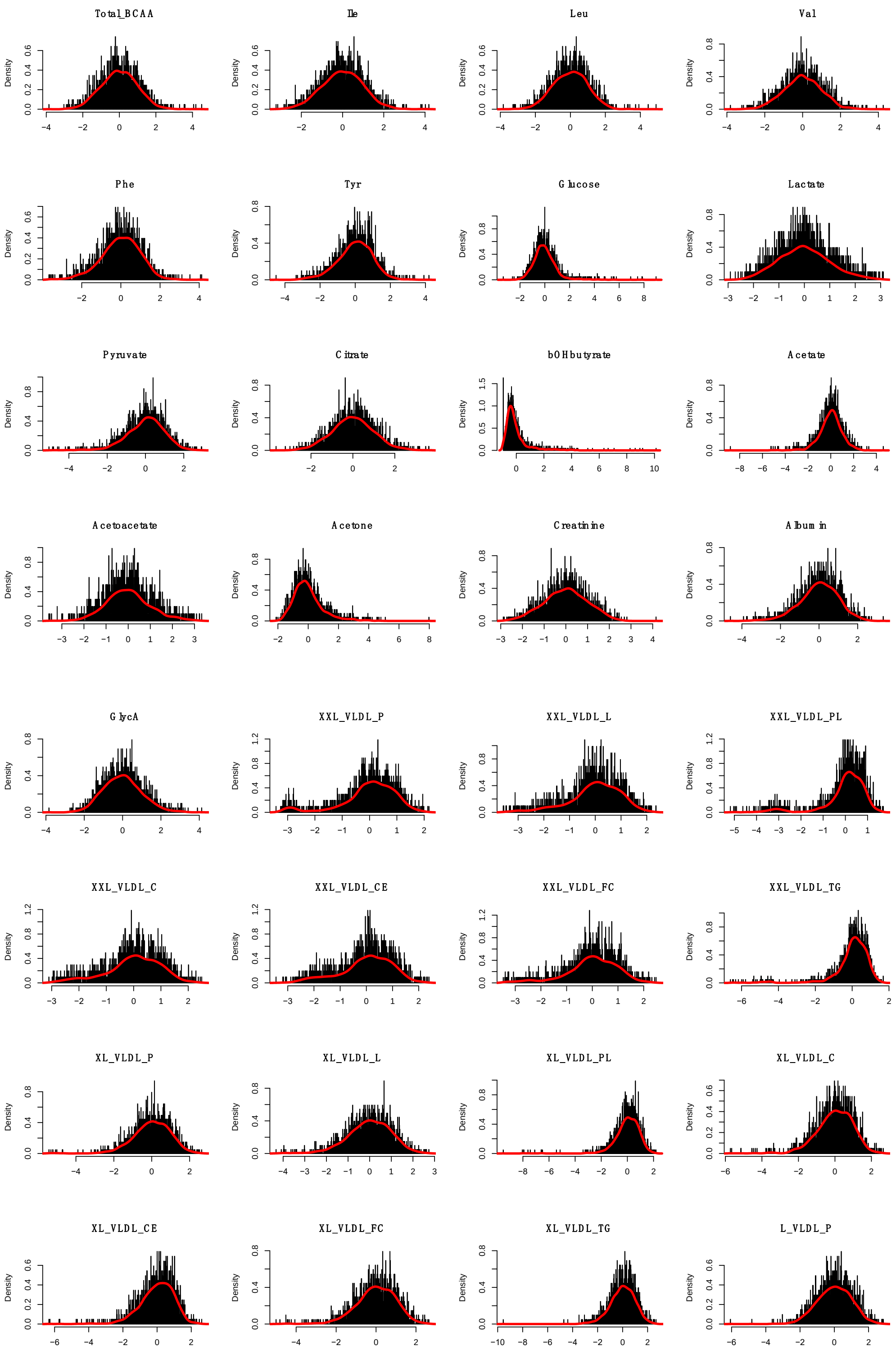

Supplemental Figure S6 Histograms of the metabolomics features after log transformation and z-scaling. Continues on the next page.

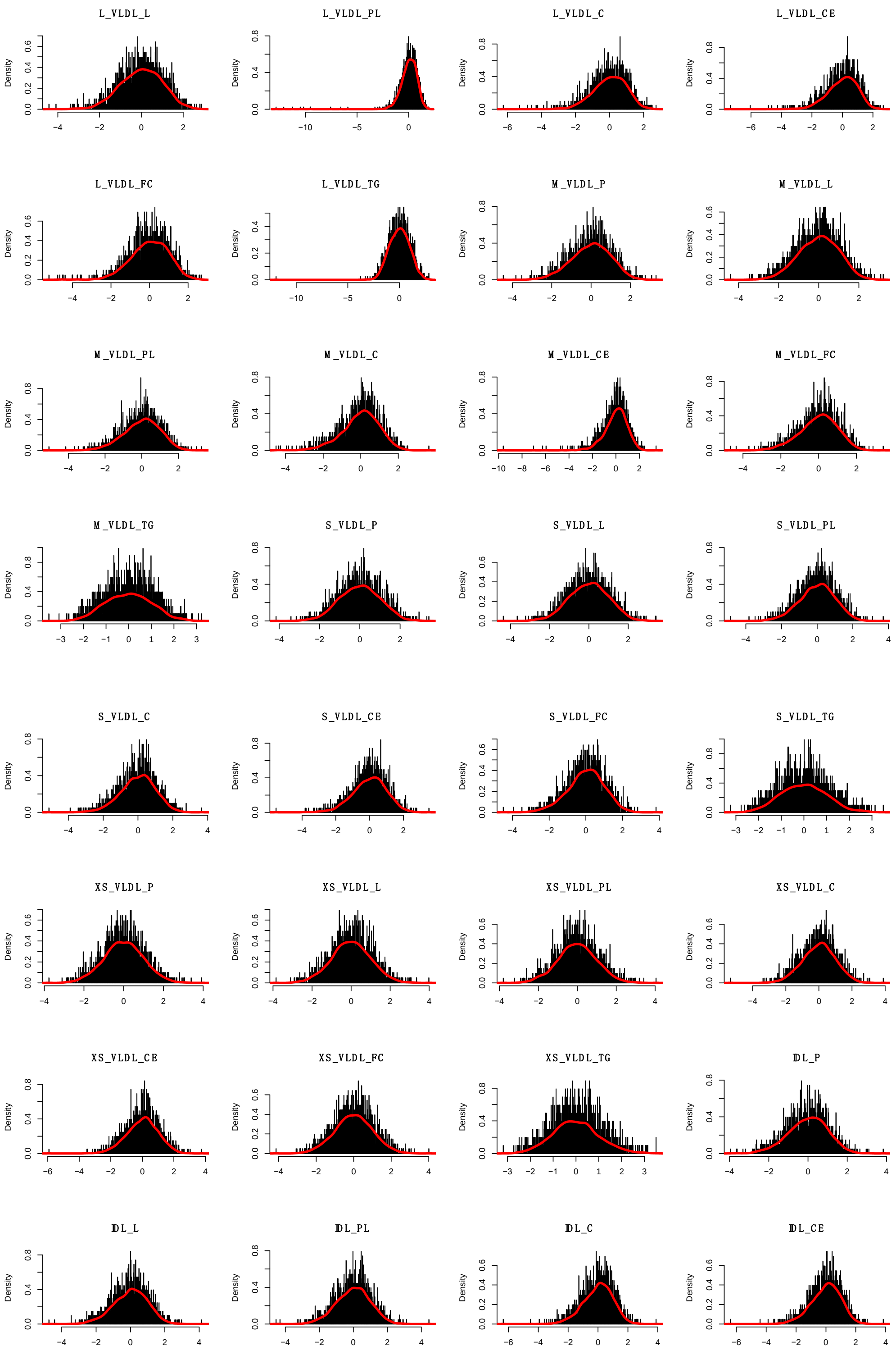

Supplemental Figure S6 Histograms of the metabolomics features after log transformation and z-scaling. Continues on the next page.

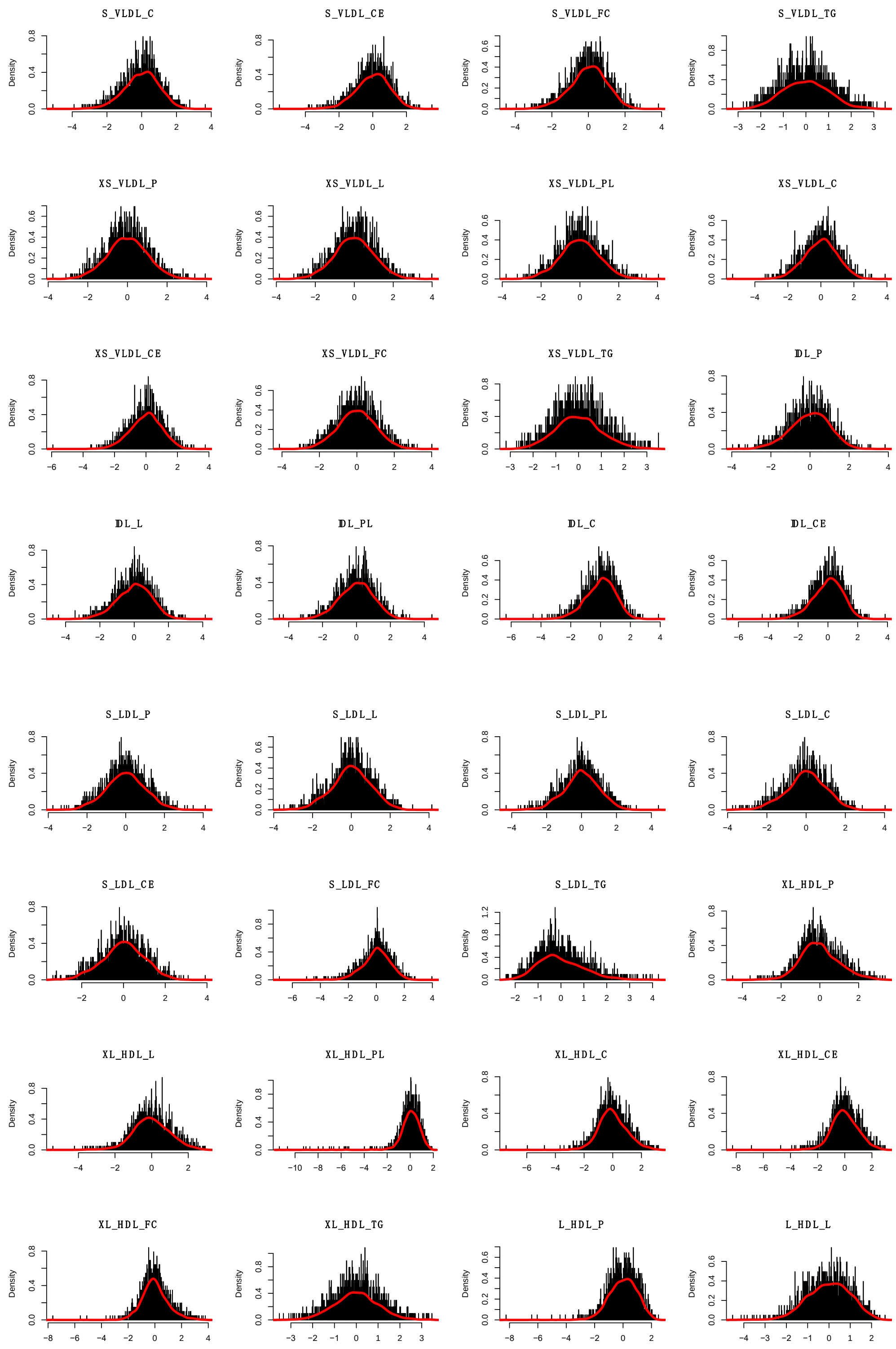

Supplemental Figure S6 Histograms of the metabolomics features after log transformation and z-scaling. Continues on the next page.

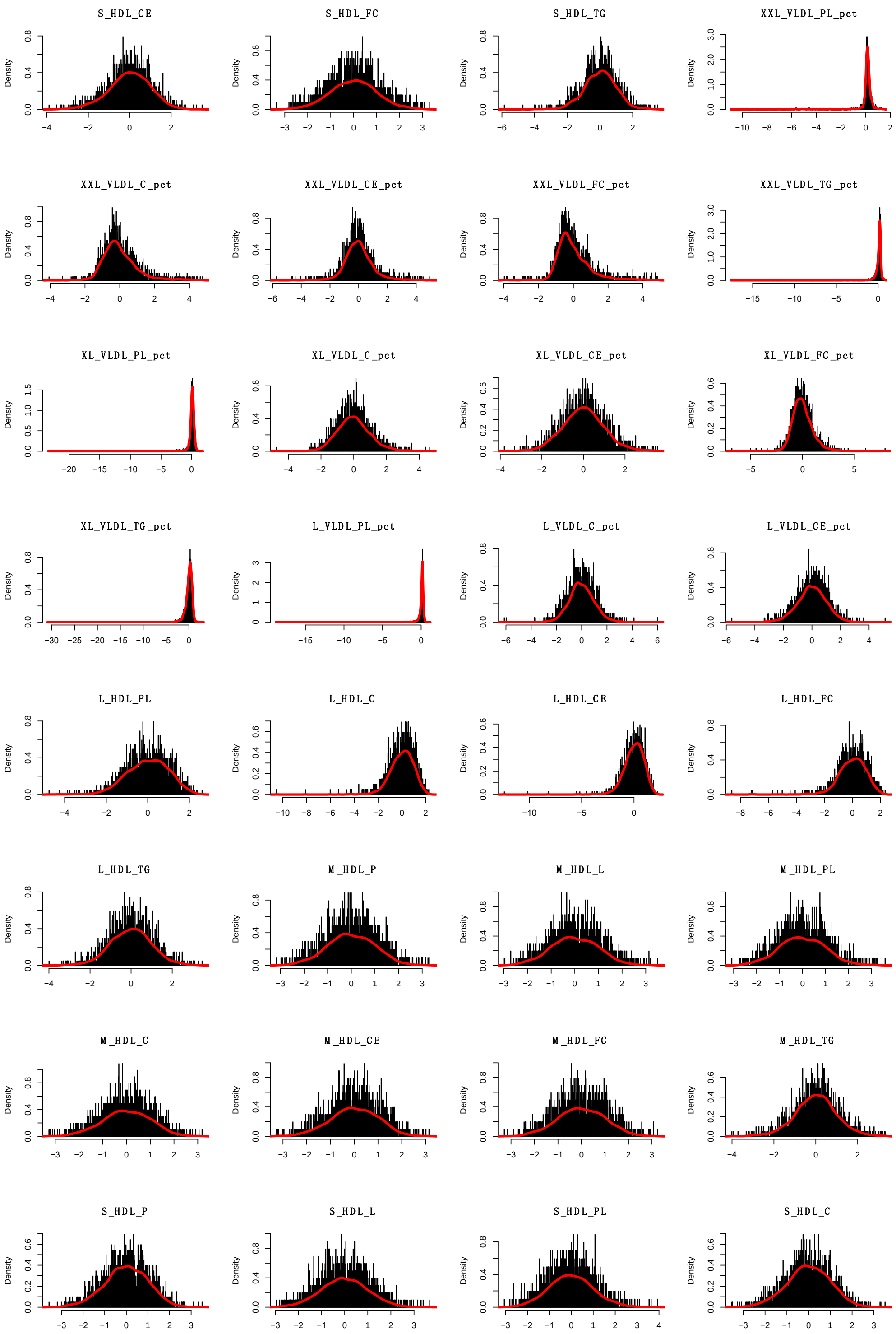

Supplemental Figure S6 Histograms of the metabolomics features after log transformation and z-scaling. Continues on the next page.

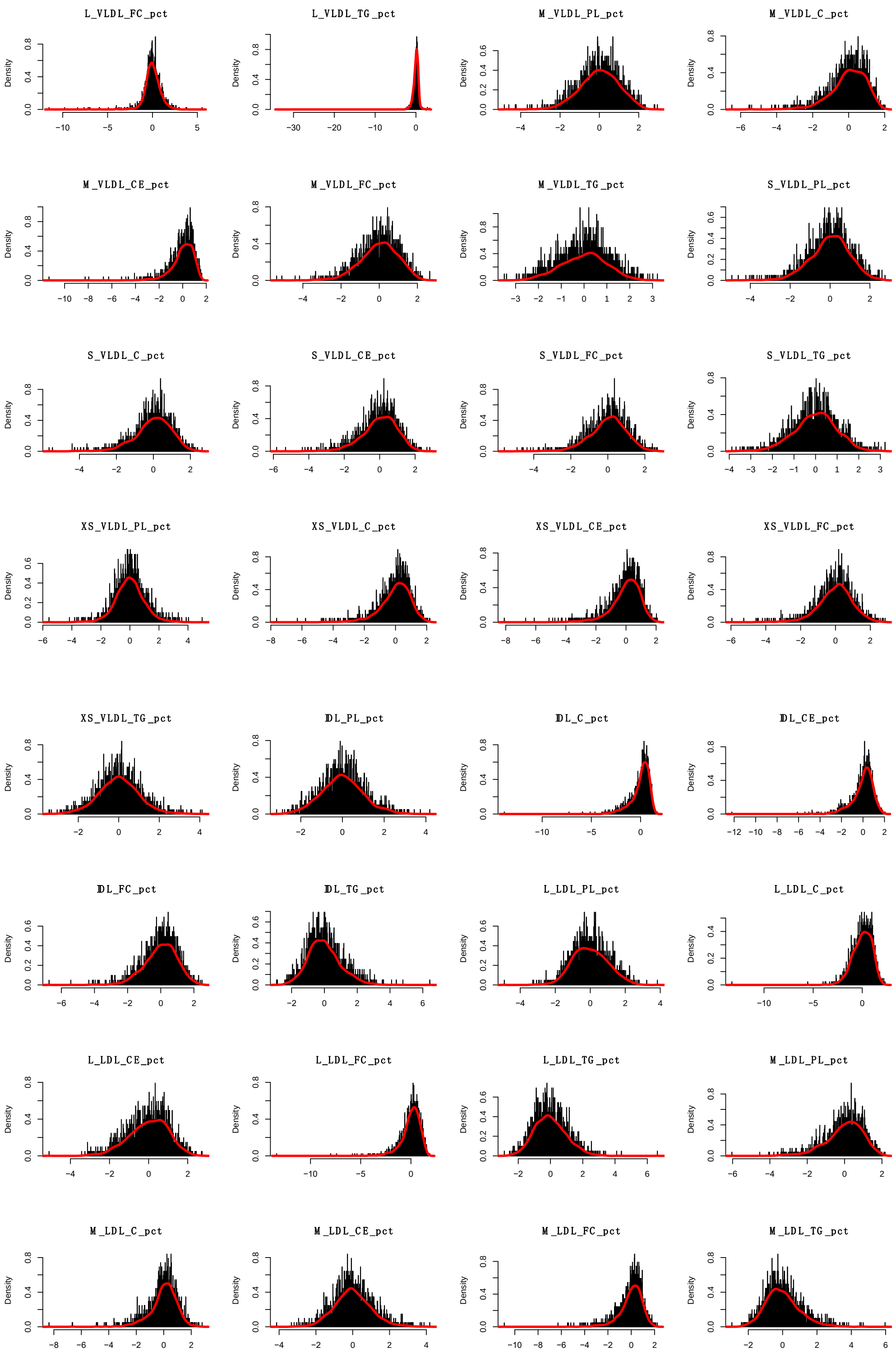

Supplemental Figure S6 Histograms of the metabolomics features after log transformation and z-scaling. Continues on the next page.

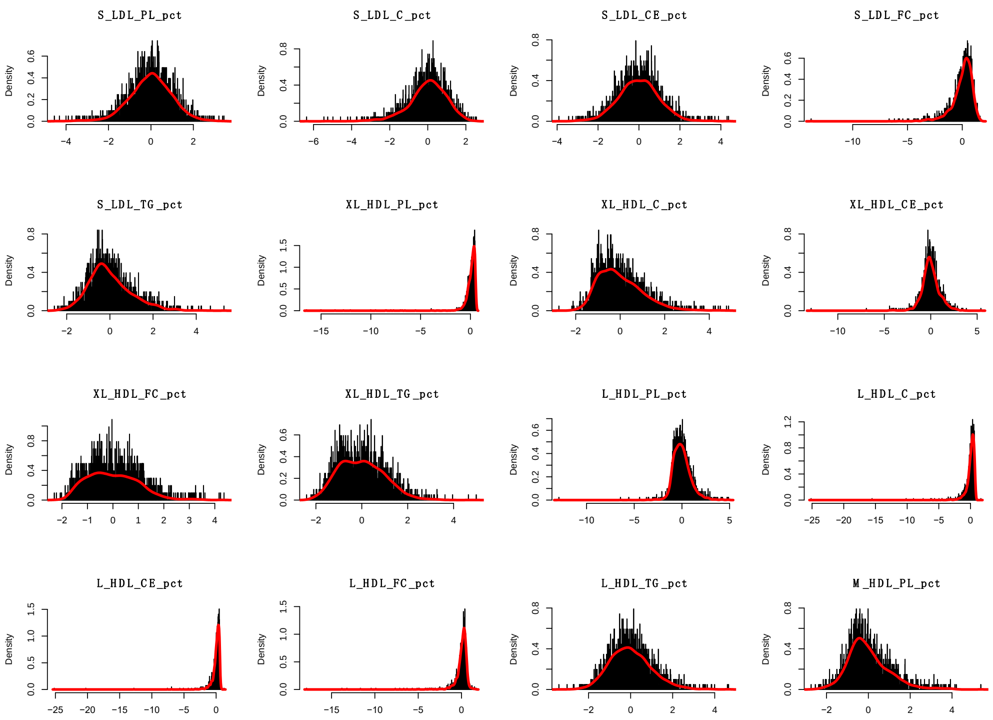

Supplemental Figure S6 Histograms of the metabolomics features after log transformation and z-scaling.

Supplemental Table S1. Disease information of the study population including stratification for sex

**Study population**	**Non-shift workers (n = 841-1008)**	**Night shift workers (n = 891-1007)**
Diabetes mellitus type 2 (yes, %)	1.8	1.5
Ever hypertension (yes, %)	21.4	21.3
Ever high cholesterol (yes, %)	11.6	11.4
**Men**	**Non-shift workers (n = 425-478)**	**Night shift workers (n = 452-484)**
Diabetes mellitus type 2 (yes, %)	X	X
Ever hypertension (yes, %)	21.4	21.9
Ever high cholesterol (yes, %)	14.9	16.3
**Women**	**Non-shift workers (n = 416-434)**	**Night shift workers (n = 421-439)**
Diabetes mellitus type 2 (yes, %)	X	X
Ever hypertension (yes, %)	21.4	20.7
Ever high cholesterol (yes, %)	8.1	5.7

Diabetes mellitus type 2: assessed at baseline and/or at the second assessment
Ever hypertension and high cholesterol: assessed at baseline
X: data not shown, because n<10 in one or more cells
